# Biofabrication approaches and regulatory framework of metastatic tumor‐on‐a‐chip models for precision oncology

**DOI:** 10.1002/med.21914

**Published:** 2022-06-16

**Authors:** Daniel Nieto, Gema Jiménez, Lorenzo Moroni, Elena López‐Ruiz, Patricia Gálvez‐Martín, Juan Antonio Marchal

**Affiliations:** ^1^ Complex Tissue Regeneration Department, MERLN Institute for Technology Inspired Regenerative Medicine University of Maastricht Universiteitssingel Maastricht The Netherlands; ^2^ Center for Biomedical Research (CIBM)/Biopathology and Regenerative Medicine Institute (IBIMER) University of Granada Granada Spain; ^3^ Department of Human Anatomy and Embryology University of Granada Granada Spain; ^4^ Instituto de Investigación Biosanitaria ibs.GRANADA University Hospitals of Granada‐ University of Granada Granada Spain; ^5^ Excellence Research Unit “Modeling Nature” (MNat) University of Granada Granada Spain; ^6^ Department of Health Sciences University of Jaén Jaén Spain; ^7^ R&D Human Health Bioibérica S.A.U Barcelona Spain

**Keywords:** 3D bioprinting, biofabrication, lab‐on‐a‐chip, metastasis, personalized medicine, precision oncology, regulatory framework

## Abstract

The complexity of the tumor microenvironment (TME) together with the development of the metastatic process are the main reasons for the failure of conventional anticancer treatment. In recent years, there is an increasing need to advance toward advanced in vitro models of cancer mimicking TME and simulating metastasis to understand the associated mechanisms that are still unknown, and to be able to develop personalized therapy. In this review, the commonly used alternatives and latest advances in biofabrication of tumor‐on‐chips, which allow the generation of the most sophisticated and optimized models for recapitulating the tumor process, are presented. In addition, the advances that have allowed these new models in the area of metastasis, cancer stem cells, and angiogenesis are summarized, as well as the recent integration of multiorgan‐on‐a‐chip systems to recapitulate natural metastasis and pharmacological screening against it. We also analyze, for the first time in the literature, the normative and regulatory framework in which these models could potentially be found, as well as the requirements and processes that must be fulfilled to be commercially implemented as in vitro study model. Moreover, we are focused on the possible regulatory pathways for their clinical application in precision medicine and decision making through the generation of personalized models with patient samples. In conclusion, this review highlights the synergistic combination of three‐dimensional bioprinting systems with the novel tumor/metastasis/multiorgan‐on‐a‐chip systems to generate models for both basic research and clinical applications to have devices useful for personalized oncology.

## 1 INTRODUCTION

More than 90% of cancer‐associated deaths are not caused by the primary tumor, but by metastasis and secondary tumors originating from it.^1^ Metastasis is a very complex process of which there are still many mechanisms to be understood to prevent the spread of cancer and, therefore, the high mortality rate associated with it. The biological processes involved are numerous, from the invasion of the surrounding stroma to the circulation through the bloodstream and the arrival to and invasion of the secondary tissue. As suggested by Chaffer and Weinberg, this complex process can be conceptually simplified into two phases: (i) physical translocation of the primary tumor to distant tissue and (ii) colonization and development of the secondary tumor.^1^ Following this simplified concept, two key points have been identified, namely cancer stem cells (CSCs) and vessel formation, since these cells are responsible for "escaping" from the original tumor and for moving to distant tissues through the circulatory system. From here, they will proceed to the invasion, establishment, and development of the secondary tumor.^2^

Conventional models of metastasis have achieved great successes but there are still challenges that need to be solved, especially in its initial phases, and related to the complexity of the tumor microenvironment (TME), which is not accurately represented in these models.^3^ For this reason, the role of CSCs must be taken into account and must be integrated with TME characteristics, since this tumor niche can directly affect the invasive capacity and behavior of these cells. In fact, essential factors are the composition and characteristics of the extracellular matrix (ECM), biomechanics, biochemical signals, the presence of different cell types, and the appropriate preparation of the metastatic niche.^4^, ^5^ Monoculture two‐dimensional (2D) assays are cost‐efficient and simple to use but have limited predictive competence failing on mimicking human physiology.^6^ While spheroid cultures can more accurately recapitulate disease characteristics and metabolic gradients than standard 2D cultures, they are avascular tumors models and lack the structure and complexity of vascularized tumors in vivo. The static culture condition associated with spheroid models does not recapitulate the in vivo conditions, including mechanical forces or dynamic flow, and so prevents long culture experiments which result in not precise drug toxicity and sensibility studies.^7^

Three‐dimensional (3D) bioprinting is a rapidly expanding and highly disruptive technology in the field of regenerative medicine, which can be defined as the automated creation of organ‐like or tissue structures using additive and subtractive manufacturing processes.^8^ The main challenge associated with 3D bioprinting for tissue engineering is to create a structure that replicates the physiology and anatomy of living tissues. These structures can be incorporated with cells (during or after bioprinting), with the appropriate biochemical signaling agents, to promote the self‐organization and shape maintenance of the assembled tissue construct. Recent advances in biofabrication technologies along with the development of new biomaterials have led to the generation of in vitro complex tissue models that can replicate the in vivo TME.^9^ Using such new technologies for reproducing the micro physiological systems that reconstitute tissue–tissue interfaces and the TME can expand the capabilities of organ‐on‐chip models and provide relatively low‐cost and more informative alternatives to animal studies. The development of these models, under such innovative methodologies, requires to be carried out under the standards of quality, safety, and efficacy that ensure its clinical transfer. Therefore, it is of interest to know the legal, technical, and ethical considerations in accordance with the competent health authority to be marketed.

In this review, we describe the advancement in the development, study, and use of tumor‐on‐a‐chip models, focusing on the metastatic process. For this purpose, we analyze the biofabrication methodologies for the generation of these models, exploring the several tumor/metastasis/multiorgan‐on‐a‐chip prototypes, and highlighting the knowledge generated related to the metastasis process thanks to these models. Finally, as a differentiating, novel, and relevant feature with respect to the rest of the literature, this review includes extensive analysis and discussion of the requirements and regulatory framework for their commercialization or use in the clinic and production, standardization, and scaling processes.

## 2 BIOFABRICATION METHODOLOGIES

**Figure 1:**
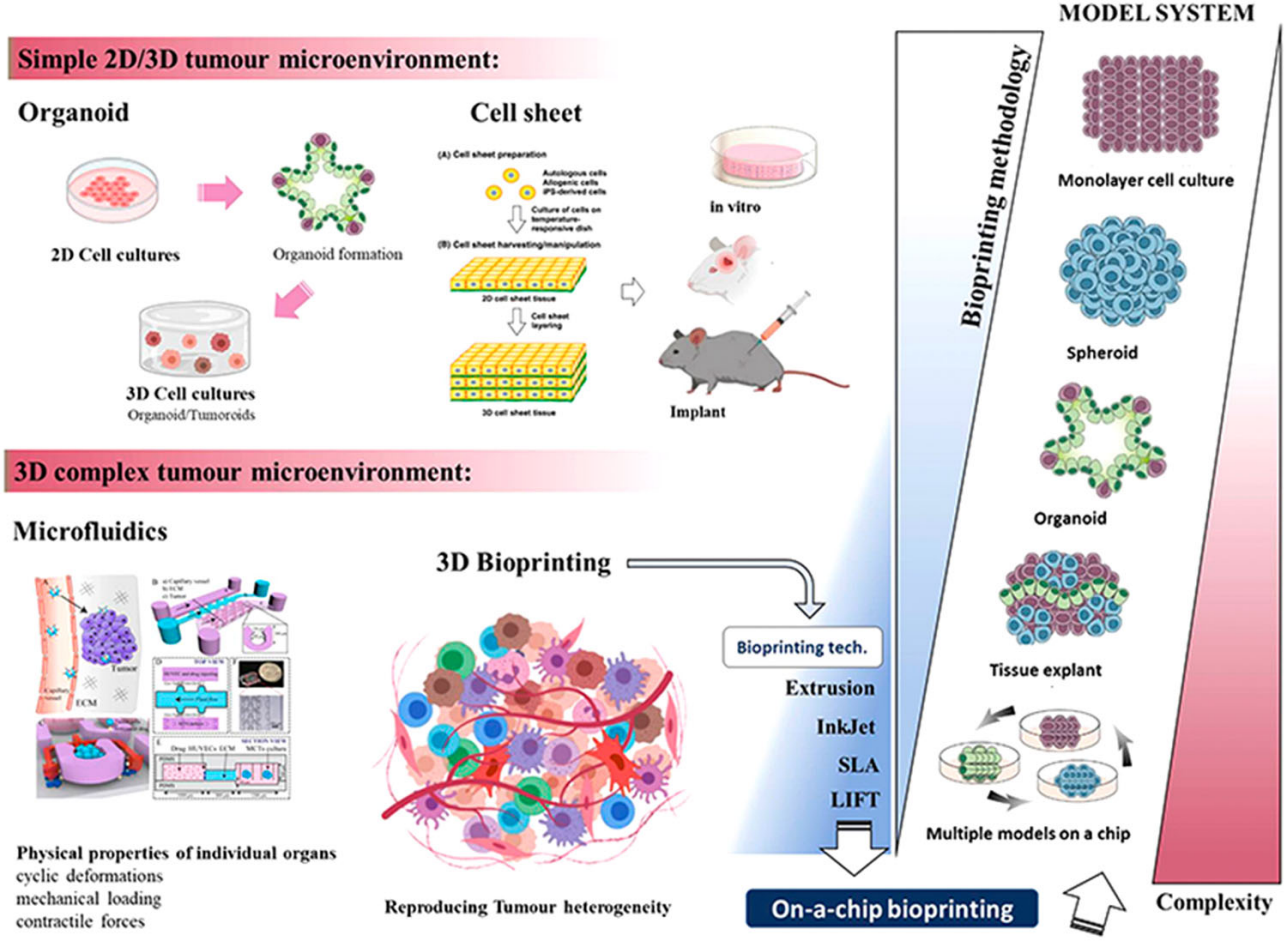
Diagram of the different biofabrication approaches for metastatic tumor model development in vitro. [Color figure can be viewed at wileyonlinelibrary.com]

Monocultures 2D assays and spheroid cultures lack the ability of recreating the structure and complexity of vascularized tumors in vivo. Such intrinsic limitations have driven the development of 3D models that can reproduce the tissue microenvironment and the associated physiology of living tissue. Current on‐chip 3D approaches mainly focus on combining various cell types, mimicking the organ components, usually using a biomaterial for supporting cell growth but also for mimicking the tissue morphology and mechanical properties. The physical properties associated to some organs can also be replicated on‐a‐chip by applying different physical stimulation which includes mechanical force to replicate the forces associated with the musculoskeletal tissue; contractile force as in the case of heart tissue or cyclic deformation for a lung model and peristaltic motion for intestine (Figure 1). A variety of organ‐on‐a‐chip (OoC) systems have been demonstrated for lung,^10^ liver,^11^ intestine,^12^ kidney,^13^ brain,^14^ heart,^15^ and musculoskeletal system.^16^

The potential of generating transplantable tissue structures using 3D bioprinting technologies has grown with the development of new biomaterials and bioprinting processes, which was demonstrated in various tissue structures including vascular grafts,^17^ nerve grafts,^18^ trachea,^19^ muscles,^20^ bone,^21^ cartilage,^22^ heart tissue,^23^ and thyroid.^24^ The incorporation of these biofabricated tissue structures into microfluidic biochips has evolved to a new area of interest, called “organs‐on‐a‐chip,” which has added a new level of complexity for modeling living organs in vitro (Figure 1). Recent studies demonstrate the great potential of OoC as in vitro drug screening systems, thanks to their more biomimetic recreation of the tissue microenvironment.^25^ Another promising application of combining 3D bioprinting and OoC is the possibility of recreating 3D‐tumor models on‐a‐chip, which are predicted to more accurately recreate the TME in vivo of human patients. This enables to recapitulate the appropriate tissue microenvironment composition including cell types, ECM, and other key parameters, such as vascularization, which match both the type and stage of the disease providing precise mechanistic studies and tools for personalized cancer therapeutic studies.^12^, ^26^ Recent advances in 3D bioprinting have allowed the manufacture of complex tissue structures with controlled architectures in the order of 10–50 μm. Miri et al. were able to generate biological tissue structures such as tumor angiogenesis, muscle strips, and musculoskeletal junctions by using a digital light projector (DLP) bioprinter and microfluidics with printing resolutions in the order of 10 μm.^27^ Such a tumor model, although primitive, can be used for studying the tumor progression and angiogenesis in vitro. As a result, there is a growing interest in adopting these technologies in emerging areas that need a highly organized biofabrication construction. Examples are tissue engineering and more realistic modeling of the TME that can reproduce the progression from early to late stages, invasiveness and angiogenesis, immune system evasion, epithelial‐mesenchymal transition (EMT), resistance to apoptosis and treatments, and metastasis.^28^ To reproduce the tumor microenvironment on‐a‐chip, different cell sourcing was used to mimic the proper physiology of the replicated microenvironment, that is, pericytes,^29^ fibroblast,^30^ myofibroblast, and muscle cells,^31^ mesenchymal stroma cells^32^; and in the case of the vasculature, endothelial cell using self‐organization,^33^ or assisted biofabrication.^34^ Although they can roughly reproduce the TME, they have not overcome the challenges associated with such 3D in vitro tumor models, which include oversimplified structures and limited vascularization potential. In this sense, some works have been motivated for replicating the vasculature. Zhu et al. bioprinted prevascularized tissue models with complex geometries (widths ≤ 50 μm and heights ≅ 50 μm) using a bioink (endothelial cells, gelatin methacrylate‐GelMA‐ and glycidal methacrylate‐hyaluronic acid).^35^ Also, Miri et al. used a DLP bioprinter in combination with microfluidics to generate tumor angiogenesis directly on‐a‐chip.^27^

For demonstrating the potential of generating 3D tumor models on‐a‐chip using bioprinting technologies, it is crucial to consider the practical issues of the available biofabrication approaches. The 3D bioprinting technology will affect the quality shape maintenance and cell viability of the final tissue structure.^36^ So, there are different 3D bioprinting methods with specific characteristics depending of the main use. (1) Laser direct bioprinting provides excellent resolution capabilities at high speed, but the process limits the inclusion of the cells due to the heat and contact generated with the laser light.^37^ (2) Inkjet bioprinting deposit liquid binding biomaterial using two different forms of inkjet printing: continuous inkjet (CI) and drop‐on‐demand (DOD). This technique can use multiple reservoirs with multicellular components for direct printing.^38^ Nevertheless, the main inconvenience of this technology is related to print speed and the risk of needle clogging associated with high viscosity, which can expose the cells to high shear forces, resulting in cell death. (3) Extrusion bioprinting use pressure or gravity for depositing biomaterials or bioinks. This technology is a low‐cost process, which works at a high printing speed allowing control over porosity and mechanical properties^39^ being an optimal candidate and used broadly for scaffolds generation. The extrusion also tolerates high cell density, which is of great interest for bioprinting, but presents low resolution and limited material choices where viscosity and bioprinting speed are crucial for maintaining cell viability. (4) Stereolithographic (SLA) is based on the photopolymerization of biomaterials. SLA usually uses ultraviolet (UV) light to solidify a photosensitive bioink in a layer‐by‐layer fashion to generate a 3D structure.^35^ This bioprinting process presents a high speed with high resolution, especially working with hydrogels, which also allows certain control over matrix properties; however, the main inconvenient of SLA appears when adding cells to the bioink, since the toxicity of monomer and UV radiation can affect to long term cell viability. Recent works used visible light to polymerize bioinks, which reduced the toxicity effects associated with UV light and which can damage cells. Lim et al. used a bioink containing living cells and a biomaterial in combination with visible light crosslinking using a ruthenium (Ru) and sodium persulfate (SPS) photoinitiator, which have demonstrated better cell cytocompatibility than the commonly used UV light crosslinking + I2,959 photoinitiator.^40^ The cells encapsulated in the bioink presented viability >85% after long time exposures to visible light, which highlights the potential of new visible photoinitiators to avoid the cell damage associated to UV light.^40^ Table 1 summarizes the advantages and disadvantages of currently available bioprinting technologies.

**Table 1:** Bioprinting technologies for tissue microenvironment on‐a‐chip fabrication.

Bioprinting technologies	Advantages	Disadvantages		References
	*Common disadvantages*			
Extrusion	Allows high cell density	Limited by the speed of printing for high‐throughput screening.	Non accepted industrial standard since 3D bioprinting is still evolving fails to reconstitute tissue‐tissue interfaces and the mechanical active miroenvironment fails to replicate Microstructures and organ vasculature limited by the speed of printing for high‐throughput screening	[36, 39]
	Low‐cost technology			
	Controllable porosity	Moderate resolution (≈100 μm)		
	Controllable porosity High mechanical strength			
		Moderate cell viability secondary to shear stress		
	Prints multiple bioink simultaneously			
	High resolution (50 μm)			
		Moderate cost for high‐resolution systems		
Inkjet	Multiple reservoirs	Low speed compared to other printers		[38]
	Direct incorporation of cells during printing	Noncomplex architectures		
	High‐throughput	Needle clogging at high ink viscosities and expose cells to high shear forces		
	No shear stress			
Laser‐based	Viscous or solid solution	Medium speed		[37]
	High resolution (1–50 μm)			
		Limited scalability		
	High resolution(3–300 μm)	High cost		
SLA	High cell viability	Monomer toxicity and use of ultraviolet radiation		[35]
	Easy control of matrix properties			
	Easy control of matrix properties	Poor hollow‐structure capabilities		
		Requires photo‐curable bioink		
	Fast speed			

Abbreviation: SLA, stereolithographic.

## 3 RECREATING TUMOR HETEROGENEITY‐ON‐A‐CHIP

In tumor development, a key step is the change of the surrounding stroma to a tumor stage associated with variations in the architecture, composition, and properties of the ECM.^41^ The tumor study models used to date have presented serious limitations, so tumor‐on‐a‐chip is a promising way to approach this study by combining the generation of a microvascular network, 3D matrices, and interaction of the tumor with stroma. This claim is supported by the microfluidics chip in poly (dimethylsiloxane) (PDMS) developed by the group of Gioiella et al., where they recreated for the first time in vitro processes that until then had only been observed in vivo. To study the remodeling of the ECM, epithelial cells from breast cancer and stromal cells were separated into different chambers, but with a barrier allowed them to interact. Then, real‐time monitoring of stromal ECM alteration during tumor invasion, based on overexpression of hyaluronic acid and fibronectin and remodeling of collagen fibers, was performed.^42^

As mentioned above, 3D multicellular culture and interaction with the components of the microenvironment are essential elements to establish an efficient tumor model with significant advantages over conventional models. A recent study showed the important role that fibroblasts play in tumor progression.^30^ Authors displayed a coculture system with microfluidic chips, where fibroblasts and tumor cells were separated into independent compartments (immersed in matrigel), but connected through a channel full of the medium. The results clearly demonstrated the effect that these fibroblasts exert on tumor development, supporting the proliferation of tumor cells and resistance to treatment. In addition, this microchip showed that fibroblasts were also influenced by tumor cells, acquiring a migratory capacity, due to their reciprocal interaction.^30^

Another relevant feature of TME is the recruitment of immune cells. To this end, the assay developed by Aung et al. describes the design of a perfusable multicellular chip where breast cancer cells, monocytes, and endothelial cells, forming an endothelial layer around them, were immersed in a GelMA with a controlled arrangement through the use of 3D photo patterns. T‐cells were included in the perfused medium and it was observed how the tumor cells favored the recruitment of these cells from the immune system.^43^ This study revealed the powerful tool that tumor‐on‐a‐chip represents to recreate the TME more precisely, modulating the cellular composition and spatial organization, demonstrating how cell populations are incorporated into this microenvironment, and providing a study tool for future therapies based on immunotherapies.

As the development of tumor‐on‐a‐chip advances, this novel technology acquires more and more complexity as demonstrated by the development of a complex 3D microfluidic chip model that emulates the colorectal TME with the ability to evaluate response to treatment in real‐time.^44^ This chip was made of PDMS with a matrigel‐embedded colorectal cancer tumor cells core that was supported by an adjacent microvascular network fed by external syringe pumps. This microvascular network originated because the endothelial cells proliferated, then elongated by the flow direction, covering the channels and resulting in a vessel‐like structure. The cells react to vascular endothelial growth factor in the hydrogel and invade the chamber by sorting the pillars. To check the real‐time monitoring, a drug‐loaded nanoparticle gradient was administered, and live images were taken to confirm the effect. In addition, the chip also allowed the analysis of gene expression, so this added to the real‐time monitoring and the formation of a microvascular network that mimicked the one generated in the TME. Moreover, this tumor‐on‐a‐chip model integrated a monitoring system allowing studies to be carried out on the same chip from treatment response analysis to angiogenesis and metastasis assays.^44^

The recreation of the TME is not only based on the cellular components, but the ECM is also a key factor to take into account since it is fundamental for the regulation of the behavior of cancer cells and it is involved in different tumor processes including metastasis. For this reason, it has recently been tried to include the components of decellularized matrices of different origins, from fresh tumor tissue, from healthy tissue, or from cell cultures in 3D tumor models.^45^, ^46^, ^47^ This advance in the recreation of TME based on the use of decellularized matrices has already been incorporated into on‐a‐chip technology, as demonstrated by a study based on a PDMS chip in which a GelMA hydrogel with a decellularized matrix of liver composed of structural proteins and growth factors was used to recreate a liver tumor, which also mimics biophysical cues such as stiffness and shear stress. This tumor‐on‐a‐chip highlights the fundamental role played by the ECM, since, unlike GelMA alone, it presented greater cellular viability and functionality, and a more specific response to treatment.^48^

Apart from the cellular components and the ECM, there are also important characteristics of TME such as hypoxia that must be taken into account when developing a biomimetic tumor‐on‐a‐chip. In this sense, the design of a PDMS microfluidic chip has been reported, which consists of a central chamber in which glioblastoma tumor cells are embedded in a collagen hydrogel and two lateral channels for perfusion of the medium, and the diffusion of oxygen is blocked by an embedded sheet of polymethylmethacrylate (PMMA). This oxygen control system was verified since the chips that incorporated the PMMA sheet presented lower levels of oxygen and pH without affecting cell viability, which changed their metabolism toward a more glycolytic behavior. This platform opens up new ways to study the effect of hypoxia on both tumor development and response to treatment.^49^

## 4 METASTATIC TUMOR MODELS‐ON‐A‐CHIP

Most deaths are due to the development of a tumor process. Most deaths are caused by secondary tumors initiated by metastasis.^1^ This is a complex process that needs to be understood to prevent the spread of cancer cells for a primary to a secondary tumor. Fortunately, novel strategies based on on‐a‐chip systems are being developed that will make it easier to uncover the remaining unknowns of metastasis, as well as pharmacological studies directed against it.

A fundamental and critical step in the metastatic process is the invasion of the surrounding stroma by the tumor. Two independent studies have designed a microfluidic chip system that simulates and controls multiple factors of the TME for the 3D evaluation of tumor invasion in the stroma. For this purpose, two independent chambers were arranged with breast cancer tumor cells in one and noncancer cells in the other, and an endothelial channel was also constructed behind the stromal chamber to simulate the microvascular network. The results showed that only a small number of cancer cells migrated through the stroma. The density of the cancer cells determined the likelihood of metastatic cells and the induction of normal cells affected the metastatic rate of each cancer cell. In addition, high secretion of interleukin‐6 by normal cells was directly related to this invasive capacity of tumor cells.^50^, ^51^

Another key step in metastasis is the process of extravasation and establishment of the secondary tumor. Tumor‐on‐chips based on microfluidic systems also allow obtaining a metastatic model of a tumor, which lets establishing the secondary tumor from two points of view: (i) the biology behind the formation of the secondary tumor and (ii) the therapeutic effect against cell migration and the establishment of tumor cells in the target organ.^52^

To address the extravasation process, a work based on a microfluidic chip in which three parallel channels were modeled in an agarose gel membrane, and chemical gradients of C‐X‐C motif chemokine 12 (CXCL12), and epidermal growth factor (EGF) were generated. By using a microfluidics platform it was show how the breast cancer cells can migrate to a collagen gel site through a monolayer of endothelial cells. The properties of the used matrix (stiffness and morphology) the interstitial flow, EGF, and the capability of regulating of CXCL12, result in a precise and controlled extravasation process.^53^ In this sense, breast and bone cancer models have been among the first to be developed and the most proliferative, because breast cancer leads to bone metastasis with a very high frequency (70%). Of the first models established, Bersini et al. developed a PDMS‐based microfluidic system in which they generated a bone microenvironment with osteoblasts and an endothelial network. It was observed that the C‐X‐C Motif Chemokine Ligand 5 (CXCL5) secreted by bone tissue plays a fundamental role in extravasation and migration through the C‐X‐C Motif Chemokine Receptor 2 (CXCR2) of breast tumor cells. In addition, these same authors subsequently demonstrated that the extravasation capacity and micrometastatic formation of circulating tumor cells (CTCs) toward the bone differed according to the origin of the primary tumor, being higher in bladder cancer, then, in breast cancer, and finally in ovarian cancer, corresponding with clinical data. Hence, the importance of the interaction between these CTCs and the secondary tissue in the success of the process of extravasation and metastasis is evident.^54^ In a more recent study, a PDMS chip was developed by printing two chambers, an upper one for medium deposit and a lower one for cell growth. After 2 weeks, they observed a behavior similar to that described in clinical cancer metastasis, which was lacking in the in vitro models developed until now. Tumor cells entered into a state of quiescence or dormancy. In addition, when tumor cells invaded bone tissue, there was an erosion of the matrix followed by an alignment of the collagen fibers with a cell proliferation adjacent to these reorganized fibers, highlighting the importance of the generation of an adequate TME for the establishment of metastasis.^55^

A key factor when developing in vitro study models is that they offer reliable results in treatment response studies. Tumor‐on‐a‐chip based on microfluidic systems, in general, has been shown to present greater resistance to treatments than conventional study models, such as the microfluidic model recently developed by Azimi et al.^56^ For example, in the work previously described, in which high secretion of interleukin‐6 by normal cells was directly related to the invasive capacity of cancer cells, response to treatment with paclitaxel and tamoxifen were also performed. The results show that the treatment inhibited the migration capacity of tumor cells being dependent on drug concentration and cell density.^50^ Wang et al. have established a model with a direct application in the prediction of the efficacy of the treatment and the evaluation of the response to different doses in the diverse stages of the tumor, showing a linear relationship of the concentration of cancer cells and the required drug concentration.^57^ Further studies are also emerging that recreate a more biomimetic TME to study the response to treatment in a more realistic way. In this sense, in the study developed by Lu et al. decellularized liver matrix was used together with GelMa in a microfluidic chip, where a better recapitulation of TME was produced thanks to the preservation of components and biochemical and biophysical signals, which resulted in a more accurate in vitro pharmacological response to the acetaminophen and sorafenib treatment.^48^ For these reasons, a microfluidic‐based chip device was a very useful tool for the efficacy determination of antimetastatic drugs.

The use of tumor/metastasis‐on‐a‐chip is not limited to the studies described so far, but the potential of this device will allow the application of personalized medicine thanks to ex vivo models of patients. They will recapitulate the specific characteristics from a small tumor sample helping to determine individually the most appropriate treatment. The first steps in this direction are already being taken, as shown by the generation of a bioprinted glioblastoma tumor‐on‐a‐chip based on patient‐derived tumor cells covered by endothelial cells as a vascular barrier, and decellularized matrix from brain tissue, all this integrated in a concentric way in a chip compartmentalized with an oxygen gradient with central hypoxia as in native tumors. This in vitro model reproduced the specific tumor resistances observed in the clinic in the patient when being treated with concurrent chemoradiation and temozolomide, so this model is very important for the establishment of a personalized treatment.^58^

In Table 2 are shown several models of metastasis‐on‐chip and multiorgan‐on‐a‐chip recently developed including the biomimetic tissues studied and their main characteristics.

**Table 2:** Metastasis‐on‐chip and multiorgan on‐a‐chip models and related biofabrication techniques.

Organ/tumor model	Bioprinting	Biofabrication technique	Comments		References	
*Metastasis on‐a‐chip*						
Blood/lymphatic vessel pair	Tumour chip with communicated environments	Extrusion/microfluidics	A tumor‐on‐a‐chip system with bioprinted blood and lymphatic vessel pair	[59]		
Carcinoma‐bone	Carcinoma‐bone metastasis model	Microfluidics bioreactor	A hepatocellular carcinoma‐bone metastasis‐on‐a‐chip model for studying thymoquinone‐loaded anticancer nanoparticles	[60]		
Kidney/liver	Liver/kidney metastasis model	Microfluidics/PDMS chamber	A microfluidic tumor‐on‐a‐chip for studying the metastasis relationship between kidney and liver	[53]		
Breast/bone	Breast cancer/bone microenvironment with osteoblasts	PDMS‐based microfluidic system	Demonstrated that the extravasation capacity and micrometastatic formation of CTCs toward the bone differed according to the origin of the primary tumor	[54, 55]		
Intestine/lung	Intestine and lung tissues on a hyaluronic acid‐based hydrogel system	Two chambers are connected in series via circulating fluid flow	Metastatic tumor foci grew in size, eventually disseminating from the intestine construct and entering circulation, subsequently reaching in the liver construct, thus mimicking some of the migratory events observed during metastasis	[61]		
Multiorgan on‐a‐chip						
Lung/brain/bone/liver	Multiorganoid chip‐based in lung cancer metastasis	Organoids/interconnected microfluidics	Invasive capacity of the tumor cells,/healthy organoids of brain, liver, and bone acquired distinctive characteristics after metastasis and invasion by cancer cells	[62]		
Colorectal cancer, liver, lung, and endothelium	Multiorganoid housed on independent chambers	Organoids/interconnected chambers	Cells migrate preferentially toward the liver and lung constructions, the corresponding organs from which more metastases of colorectal cancer arise in the clinic, constituting by both a representative in vitro system for drug screening	[63]		
Liver/cardiac/lung, vascular/testis/colon and brain	A human primary cell‐ and stem cell‐derived 3D organoid technology	On‐a‐chip platform comprised of multiple tissue organoid types	The 3D organoid system was able to demonstrate toxicity. Organoids exposed to nontoxic compounds remained viable at clinically relevant doses	[64]		

Abbreviation: 3D, three‐dimension; CTC, circulating tumor cell.

## 5 HOW CAN TUMOR‐ON‐A‐CHIP MODELS HELP WITH THE REMAINING UNKNOWNS OF THE METASTATIC PROCESS?

Although the mechanisms associated with many of the tumor processes and the fundamental role played by the different subpopulations and the TME are known today, there are still challenges associated with the study of metastasis to be resolved, especially at the initial stages, where CSCs and the tumor vasculature play a fundamental role. Therefore, it is essential to understand their role in metastasis, and that is the reason why new tumor‐on‐a‐chip models are needed to address these knowledge gaps as we will describe below.

### 5.1 Tumor‐on‐a‐chip models based on CSCs

Within the bulk of a tumor, there is rare and scarce cell subpopulations identified as CSCs that are characterized by expressing specific membrane surface markers, as well as by high aldehyde dehydrogenase 1 activity. In the same way as mesenchymal stem cells, CSCs are automatically renewed and display the expression of stemness genes. They also have the ability to evade the effect of treatment thanks to the acquisition of an inactive state of quiescence and the ability of multidrug resistance (MDR). Although today there are precise mechanisms involved in metastasis that remain to be elucidated, CSCs initiate the metastatic process and then generate a secondary tumor in a distant tissue, and they also originate tumors after primary therapy.^2^ Since the presence of this subpopulation in the TME and its fundamental role in tumor evolution are well known, efforts have been joined to understand both its intrinsic biology and to search for therapeutic alternatives against them. However, the main trouble is the difficulty to grow and maintain their stemness properties in vitro, a problem that 3D culture has gradually solved.^65^

The first investigations that integrated 3D bioprinting systems with CSCs were carried out to study the development, vascularization, and metastasis in gliomas. In these studies, extrusion bioprinters were used to bioprint CSCs loaded in hydrogels composed of gelatin, alginate, and fibrinogen as the main elements of the ECM, and the results showed consistent differences with respect to the conventional study models, such as higher (i) stemness maintenance, (ii) differentiation potential; (iii) resistance to treatments; (iv) angiogenic capacity; (v) EMT; and (vi) tumorigenic capacity.^66^, ^67^, ^68^ Therefore, this 3D tumor model represents the most suitable to investigate the biological characteristics and tumor processes in which CSCs are involved. Although these studies are based on non‐miniaturized bioprinting, they are very important because they are the first step toward the development of on‐a‐chip platforms.

Currently, tumor‐on‐a‐chip studies with CSCs are still very scarce in the bibliography, and one of the reasons for this is that these cells are difficult to maintain in culture. Regarding this handicap, a novel and very recent study develop a droplet microfluidic single‐cell culture for selective expansion and recovery of CSCs. For the development of the system, a single CSCs were incorporated into alginate hydrogels in a PDMS microfluidic chip, and thanks to the self‐renewal property of these cells, tumor spheres were formed from which the cells could be recovered later. This novel system provides a sufficient quantity of CSCs for both the study of CSC biology and the development of new therapies.^69^

Focusing on studies that analyze the characteristics of CSCs using microfluidic chips, one of the first studies dates back to 2016 and was developed by Yahara et al., where the role of oxygen in the migration of both CSCs and differentiated tumor cells was analyzed. Results showed that the migration patterns of CSCs did not differ from the differentiated tumor cells, both traveling toward low oxygen levels.^70^ Surprisingly, these results differ from a previous publication, where the cells migrated toward high levels of oxygen.^71^ This difference in results may be due to the fact that the chip had an exhaustive control of the microenvironment, only varying the oxygen concentration, while in the study without the chip there may have been external influences. Furthermore, these results are reinforced and supported by another recent study developed by Aung et. al., who showed that tumor cell cultures in spheroids presented higher levels of hypoxia than individualized cell cultures. They also observed how these spheroid cultures significantly recruited more T‐cells compared to isolated cells, thus highlighting the differential role of the different subpopulations in TME and the central role that CSCs play.^43^ But the migration of CSCs is not only influenced by oxygen concentrations, otherwise by migration under chemotaxis. In this sense, microfluidic chips are very useful to generate multiple stable and controllable gradients through a device that integrates a gradient generator module and a cell culture chamber. This chip allowed us to establish how CSCs and differentiated tumor cells migrate a greater distance in a higher serum gradient, and how under drug treatment the distances traveled decreased by 70%–80% for both cell types, constituting a novel analytical platform for the study of cell migration and drug screening.^72^

Another very important focus in the study of CSCs is resistance to treatment. For this purpose, the study recently published by Lin et al. presents a microfluidic chip that allows the isolation, identification (through its ability to form spheroids), and culture of breast CSCs, to subsequently perform high‐throughput screening to detect anticancer drugs with specificity against these breast CSCs, doing all this on the same device.^73^ Thanks to the generation of devices with these characteristics and capabilities, not only is specific pharmacological screening addressed, but also the limiting factor posed by the complex culture of CSCs, which presents an important challenge for conventional cell assays.

Also, as it has been reviewed by Lin et al.,^74^ the advances in the generation of devices focused on the isolation and analysis of CTCs have great potential as clinical decision support tools in precision oncology. These CTCs are CSCs that leave the primary tumor and intravasate into the bloodstream and lymphatic vessels and they have the potential to predict metastasis and evaluate tumor activity, being effective for the detection of relapses and with a high prognostic value.^75^ An example of the translation of these devices to the clinic is the Labyrinth microfluidic device designed by Wan et al. to detect CTCs in the blood through a process of separation at a flow rate using a syringe pump, followed by immunofluorescence staining with specific markers of CTCs and CSCs. Thanks to this device, CTCs were detected in blood in 88.1% of patients with hepatocellular carcinoma. Moreover, a correlation was established between the results and tumor stage, since more advanced stages detected more CTCs, as well as the presence of circulating tumor microemboli, thus constituting a very useful tool for analyzing tumor progression.^76^ In fact, a commercially disposable microfluidic chip called On‐Chip Sort® (On‐chip Biotechnologies) is currently available and validated for different types of cancer and it works under the principles of flow cytometry to perform a cell‐sorter that includes a collection tank to store collected CTCs.^77^, ^78^ Thanks to this small chip, CTCs can be detected and have allowed the development of a protocol for early diagnosis and monitoring of metastasis.

All these results together, open up new research approaches in the area of CSCs and the TME, in which microchips represent a new starting point compared to conventional study models.

### 5.2 Tumor‐on‐a‐chip models to optimize the tumor vasculature

Oxygen and nutrients supplied by blood vessels are required in almost each human body tissue and tumor. The TME is very complex and presents different characteristics and behavior at each stage of the disease, including the degree of vascularization, stiffness of matrix, and the ECM and cellular composition.^79^, ^80^ Then, to obtain this blood supply, tumor cells can stimulate the expression of angiogenic factors to drive vascular growth by attracting and activating cells of the TME. In comparison to healthy tissues, the tumor vessels are structurally and functionally abnormal.^81^ To understand the complexity associated with the TME, and to identify drugs to treat it, in recent years, in vitro models that accurately reproduce the human tumor physiology are emerging as a more realistic tool. It was motivated because current animal models do not faithfully reproduce the human physiology of cancer and, therefore, fail in their pharmacological response.

In this sense, some attempts to replicate vasculature on‐a‐chip have been made. Chen et al. provided a high‐resolution view of tumor cell extravasation through microvessels formed in a microfluidic device.^82^ Also, Miri et al. have generated a tumor angiogenesis on‐a‐chip using a microfluidic multimaterial bioprinter.^27^ Nevertheless, the development of a vascularized organs model on‐a‐chip requires overcoming the challenges associated with existing biofabrication techniques to generate functional and vascularized in vitro tumor models that preserve their in vivo characteristics and phenotypes, which are prerequisites for accurate biological studies and drug screening. In this sense, tumor on‐chip with associated microvascular networks can reproduce the structure, function, and any process associated to a vascularized tumor in vivo. Vascularized tumors can also be used to investigate unclear metastasis processes involving the interactions of endothelial and stromal cells with tumors. It also can be used for drug screening by investigating the relevant physiological barriers to efficient drug delivery, including antimetastatic and antiangiogenic drugs.

## 6 MULTIORGAN‐ON‐A‐CHIP: IT IS THE MOST REPRESENTATIVE MODEL?

The process of developing new drugs requires toxicity testing associated with the new compounds to verify their safety. Currently, the most widely used methodology is the ADME‐Tox technique, which consists of an in vivo test with animal models for evaluating the absorption, distribution, metabolization, and elimination of the products. However, this methodology has the inconvenience that after being approved therapeutic compounds for widespread use in humans; they have had to be subsequently withdrawn due to unforeseen toxicity. These events are largely due to flawed data generated from preclinical in vivo and in vitro models that do not accurately recapitulate human physiology. Moreover, to this must be added the high associated costs of in vivo experimentation, as well as the requirement of highly specialized personnel and facilities.^83^ Due to these problems, the idea of the creation of integrated organoid and multi‐organoid systems that contain liver, heart, lung, vascular, testicle, colon, and brain is emerging for the toxicological screening of drugs, proving to be a more physiologically relevant model. The costs of this multiorganoid platform and the failure rate associated with the approval of new drugs are expected to be reduced.^64^, ^84^, ^85^

The creation of a multiorgan‐on‐a‐chip generates the necessary technical support to advance both in the study of metastasis and in pharmacological screening techniques.^85^ The first devices that comprised more than one tissue to simulate a metastatic response to the treatment process consisted of two different pairs of tissues such as breast and bone tumor and kidney and liver described earlier in this review.^54^, ^55^, ^57^ Among the first published works that developed a tumor‐on‐a‐chip with multiple tissues is the one described by Hwan et. al. These researchers generated a chip, in which the flow of medium together with the drug to be tested was induced by gravity (bypassing a pumping system to avoid the formation of bubbles) reached up to three different chambers, where there were cells in culture that represented the tumor, liver, and the bone marrow. The response to treatment was conditioned by the cell type and by the condition established by the flow in comparison with a static environment, which is why one of the first platforms was provided to test pharmacological toxicity in an improved way, this being the first antecedent of the multiorgan‐on‐a‐chip.^86^

After these reductionist models, more complex devices have emerged in terms of cell composition/representation and chip design. Good proofs of this are the studies developed by the pioneering research group of Aleksander Skardal and Anthony Atala at the Wake Forest Institute for Regenerative Medicine. In one of their first jobs, they developed a metastasis‐on‐a‐chip device with a double chamber, where a metastatic colon carcinoma tumor and a healthy liver were recreated and connected by a microfluidic system. This novel system allowed, first, real‐time monitoring thanks to the fluorescent labeling of tumor cells, observing how these cells acquired a metastatic phenotype and migrated from the tumor to liver construction, and second an antimetastatic pharmacological test.^61^

In the latest advancements in the design of a more complex chip with the representation of multiple organoids, the work developed by Xu et al. generated a multiorganoid chip based on lung cancer metastasis that included lung cancer representation and brain, bone, and liver tissues. All these organoids were housed in independent chambers and connected by a microfluidic system. This system allowed analyzing the EMT and the invasion capacity of the tumor cells, and how the cells of the healthy organoids of the brain, liver, and bone‐acquired distinctive characteristics after metastasis and invasion by cancer cells. These results provide insights into how such a useful tool could bridge more closely the gap with the in vivo microenvironment of cancer metastasis and investigate cell–cell interactions during metastasis.^62^ In addition, one of the most recent devices has been designed by Aleman et al. and consists of four different organoids, colorectal cancer, liver, lung, and endothelium, housed in independent chambers. This system allows cell tracking through fluorescence imaging, and it was observed that colorectal cancer cells migrated preferentially to the liver and lung. These results are in line with that has been observed in the clinic, thus constituting a representative in vitro system for screening antimetastatic drugs.^63^

Finally, the recent study developed by Zheng et al. highlights the great potential of these systems by incorporating not only a multiorgan metastasis model but also recreating the role of the microenvironment in this process. On this chip, lung cancer and healthy liver cells were cultured in isolated chambers, and precise and highly controllable normoxia/hypoxia conditions were established. The hypoxia condition was shown to favor the metastasis process through EMT induced by the HIF‐1α factor and further demonstrated its potential as a pharmacological screening platform under hypoxic conditions.^87^ This novel study goes a step further, demonstrating the full potential of these platforms, in which several microenvironmental factors that were not previously recreated in conventional study models can be controlled.

## 7 REGULATORY FRAMEWORK OF TUMOR/METASTASIS/MULTIORGAN‐ON‐A‐CHIP MODELS

**Figure 2:**
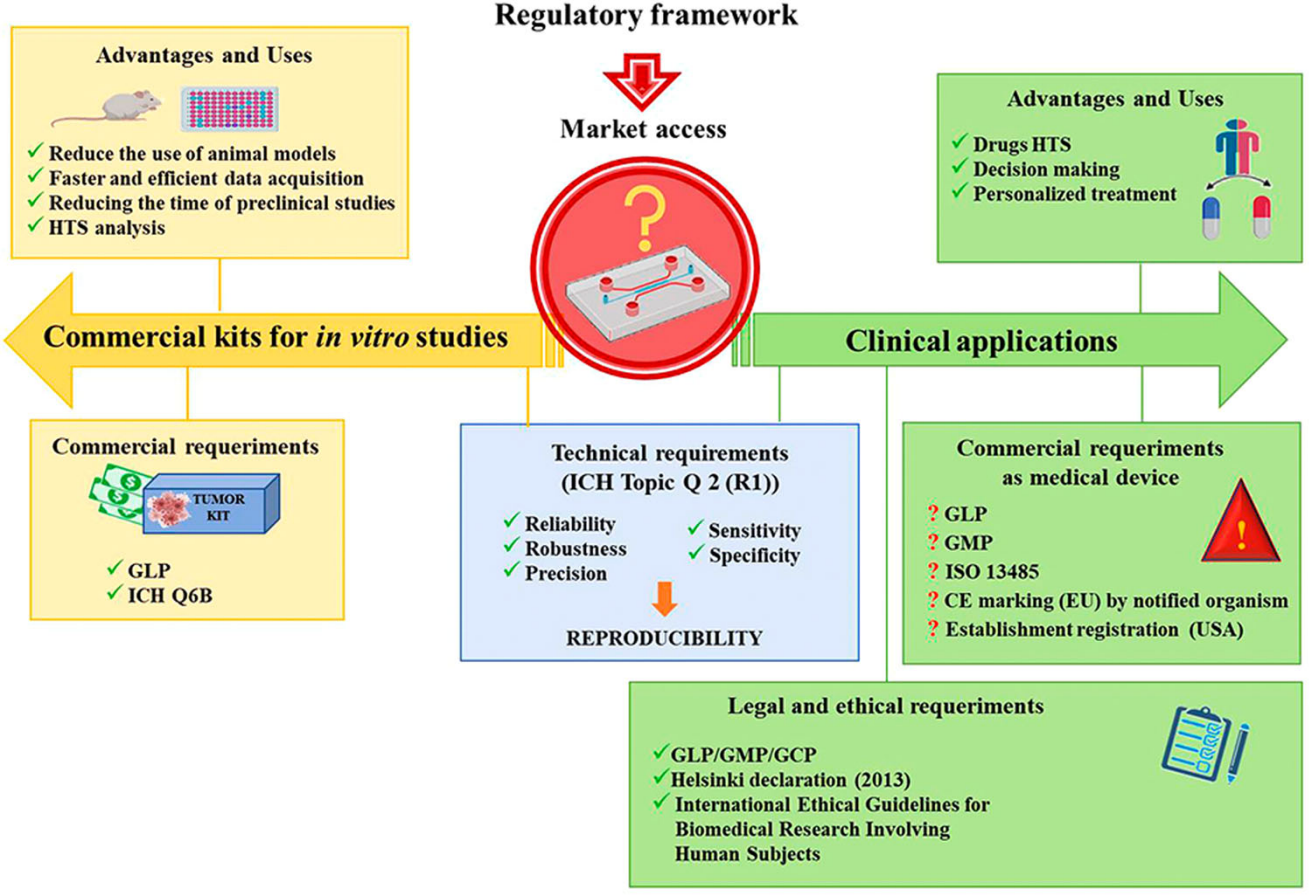
Schematic of the international regulatory guidelines addressing regularities, scientific and techniques changes needed for tumor/metastasis/multiorgan‐on‐a‐chip models commercialization and clinical use. [Color figure can be viewed at wileyonlinelibrary.com]

Currently, tumor/metastasis/multiorgan‐on‐a‐chip models and all the innovative technology involved in their progress are being widely studied to implement their use as devices for personalized medicine. The development of these models should be performed under specific technical, ethical, and legal requirements to be approved for their marketing according to their regularity framework (Figure 2). These requirements should be considered throughout their design and development to optimize their translational utilities.^88^ Thus, it is necessary to know the specific regulatory pathway for each model based on their different applications such as (i) devices for clinical use either as a diagnostic tool in routine clinical practice that allows optimizing the selection of cancer drugs or as patterns that allow ex vivo monitoring of the evolution of the tumor; and (ii) in vitro study models for preclinical research as an alternative to the animal experiments.^89^

Regarding the technical requirements of tumor/metastasis/multiorgan‐on‐a‐chip models, they should be validated for each of their uses. This phase is one of the most important, since with the validation of each model its reliability, robustness, precision, sensitivity, and specificity are evaluated to ensure its reproducibility every time a model is biofabricated.^90^ Thus, after the validation process, a range of conditions of use is determined, providing reproducible and accurate data for each type of model. The aim of validation is to elaborate conformity assessment procedures, which will be evaluated by the corresponding healthy authorities. These studies can follow the recommendations described by the International Conference on Harmonization (ICH) guidelines, “ICH Topic Q 2 (R1) Validation of Analytical Procedures: Text and Methodology” (Figure 2).

On the other hand, it is necessary to define the analytical quality specifications for each type of on‐a‐chip model. These specifications describe the appropriate technical and commercial acceptance criteria that each type of on‐a‐chip model must satisfy for its approval for the healthy authority (to be marketed) and for the customer (to be used correctly). For these studies, the “ICH Q6B Specifications: Test procedures and acceptance criteria for biotechnological/biological products” guideline set the principles to establish international specifications for different biological products with tumor/metastasis/multiorgan‐on‐a‐chip models‐like characteristics. In addition, the manufacture of these devices should be performed under the standards described in Good Laboratory Practice (GLP) guidelines^91^, ^92^ (Figure 2).

It is necessary to emphasize that the collection, processing, in vitro culture, and storage of human biological samples (biopsies) to obtain CSCs from which to biofabricate tumor/metastasis/multiorgan‐on‐a‐chip models should be performed under the ethical principle described in ethical internationally recognized guidelines such as the Declaration the Helsinki (2013), the Nuremberg Code or the International Ethical Guidelines for Biomedical Research Involving Human Subjects.^93^, ^94^, ^95^ Moreover, the approval of these procedures by an expert committee should be required to evaluate the ethical acceptability of use for each of on‐a‐chip model according to its final use. Thus, an informer consent of the patient (for models with autologous cells) or donor (in the cases of the use of allogenic cells) will also be necessary^96^ (Figure 2).

Concerning the on‐a‐chip models as devices for clinical use, approval should be required by the health authorities. Thus, each on‐a‐chip model should meet specific legal requirements according to the jurisdiction where the product is going to be used ensuring they are safe and perform as intended. Therefore, to know what the legal aspects are necessary to be able to follow the adequate regulatory pathway for their commercialization should be considered. However, due to their innovative nature, there is currently no defined regulatory framework for them, since they would be outside the definition of medicine, advanced therapy medicinal products, medical devices, and in vitro diagnostic products.^97^, ^98^, ^99^ Hence, the legal status of the tumor/metastasis/multiorgan‐on‐a‐chip models has not yet been defined. For the moment, it will be necessary to request the classification of these devices by the competent health agencies. In addition, the health authority should define if it is a notification or premarket approval. In the case that a premarket approval should be required, it is the responsibility of the competent health authorities to evaluate the technical and ethical documentation provided, from which the devices should be authorized or not for their marketing. All of them imply that the marketing request for each type of on‐a‐chip model requires that it be analyzed case‐by‐case until they are officially regulated under a specific regulation.

According to current legislation, the most appropriate consideration for these models would be in vitro diagnostic products or medical devices. Thus, it is expected that for the marketing of an on‐a‐chip model in the European Union should be necessary to undergo a conformity assessment and apply conformité européenne marking in an accredited notified body. In the case of the United States, an establishment registration in Food and Drug Administration should be required^100^ (Figure 2).

The great advances that are being made in the development of these models should have an impact on international regulatory guidelines addressing regularities, scientific, and techniques changes. These changes will stimulate the implementation of specific requirements for this type of innovative product. All of them will entail the need to involve the regulatory agencies in the development of these models and to work together to define the legal requirements that will be necessary to be authorized.

These models will become an integral part of the safety and efficacy evaluation processes either as a commercial kit for in vitro studies or as devices for clinical use. On the one hand, the applications of these models in preclinical studies will lead to accelerating the collection of safety and efficacy data, which will be able to support clinical trial authorization applications of the evaluated drug. On the other hand, tumor/metastasis/multiorgan‐on‐a‐chip models represent a breakthrough in the testing of new cancer drugs and in selecting the most suitable therapy for each patient. In addition, the use of these models will allow designing new procedures for the study of new active ingredients and medicines in the phase of preclinical research.

## 8 FUTURE DIRECTIONS IN STANDARDIZATION AND SCALABLE PRODUCTION

The tumor on‐a‐chip model is a promising and potent tool to study cancer progression and metastases. The physiological, chemical and mechanical influence can be analyzed with the incorporation of heterogeneous bioprinted tumor models on‐a‐chip52. Pharmaceutical companies and clinicians require high throughout the put fabrication process to facilitate optimal differentiation, generate multiple tissues and organs, or multiorgan systems to investigate new therapies and drug compounds. In this sense, future work needs multidisciplinary research on modeling and simulation, bioengineering, bioprinting, biofabrication, and biomaterials. Although such biofabrication technologies are expected to transform the diagnosis and treatment for a broad range of medical conditions, there is a burning need to close the gap between the clinical needs and the continuing demand for the scientific gain of knowledge, which slows down the translation of real solutions to the clinical arena.^101^

Establishing new protocols to control the use of multiple biomaterials and cells will be a key point to rebuild complex tissue structures and interfaces. The amount of biomaterials available for biofabricating synthetic tissues is currently very limited. In terms of manufacturing the key issue is to maintain good processability while minimizing their impact on cell viability.^102^ New biomaterials development needs to evolve in parallel with bioprinting technologies and with more effective reactive biomolecular components (e.g., growth factors) to better control and influence cell activity. Such tasks will require overcoming the limitations of current biofabrication protocols, which include better engineering of desired mechanical properties, and improving resolution and bioactivity with elasticity profiles. Establishing good manufacturing practices (GMP) and GLPs for biofabrication tumor/metastasis on‐a‐chip at all process levels to ensure the abovementioned technical standards will ensure a rapid translation. Moreover, a better comprehensive framework of biofabrication and biomanufacturing technologies is necessary to generate market‐ready tissue models on‐a‐chip. This will improve repeatability, a critical point for standardization, and multimaterial handling capabilities. Such novel technologies will also push the existing materials toward new properties and capabilities. Innovative additive biofabrication hardware solutions (e.g., printheads, high‐resolution stereolithography systems, microfluidics, bioreactors) generated as new devices will allow diversifying in choices of biomaterials for new generation biofabricated products.^103^, ^104^, ^105^

In this sense, digital manufacturing has the potential to disrupt and transform medical device manufacturing based on additive and subtractive processing of biomaterials and bio inks, while in a “GMP‐ready” environment.^106^ The challenge of efficiently realizing new treatment methodologies, and accelerating the introduction of more complex and biocompatible medical devices, requires better insights into how materials and cells respond to each other and to physical stimuli. This ambition depends on the future ability of scientists and engineers to exploit scalable digital production of smart on‐a‐chip systems tailored to the needs of individuals in a future low cost, advanced manufacturing environment.

The future vision of digital manufacturing to realize high throughput on‐a‐chip products is to integrate functionalities attained for printed electronics in medical device technology. Printed flexible electronics have realized capacitive touch sensors, organic light‐emitting diodes, thermal heaters, photovoltaics, smart windows, high density interconnects, and many other sensors. This technology is ready to move to the biomedical sector with the opportunity to span the following target areas: (i) realization of new bio‐diagnostics/microreactors based on microfluidics; (ii) development of new tissue‐engineered structures for cell viability, therapeutic tests, and future OoC applications; (iii) the tissue engineering activity is also relevant to wound care, wearables, smart implants with wireless interfaces, passive energy sources, and drug delivery functionalities.

Systems based on smart polymers displaying photo‐responsive properties could benefit from the integration of printable light‐emitting materials. Recently, drug delivery systems utilizing optically activated nanocarriers have also received extensive attention. A directly relevant example to this vision is the Pilot‐line for Bio‐Microsystems at the National University of Ireland, which aims to create additive and subtractive manufacturing testbed for creating next‐generation electrically, optically, and thermally activated biomaterials.^108^ The integration of nanoparticle quantum dot fluorescent labels with printed tissue scaffolds could use such as a source to assess local pH and O_2_ content in printed tissue scaffolds.^107^, ^108^ Microfluidic platforms exploiting incorporating a microheater have been designed for the study of the metastatic potential of lung cells.^110^ Developing technologies for the study and control of cell behavior under an applied electric field is another application for future biofabrication requirements. Previous results indicate that such electrical stimulation can improve alignment, elongation, and proliferation of cells.^111^

The grade of innovation and the complexity, which is characteristic of biofabrication technologies and the resulting novel devices and products generated with them, need to struggle with the natural opposition in some sections of the market. Such classic skepticism underlines the importance of generating effective public engagement protocols to get industry and end‐users involved first in understanding the need for such complexity and, then, to help on pushing these technologies toward societal acceptance and use.^112^ In this direction, ethical considerations need to focus on ensuring the sustainability of biomaterials, cell sourcing, and biofabrication of tumor models and making sure the benefits are universally available.

Regulatory approval processes involving biomaterials, tumor on‐a‐chip, and tissue constructs, and related protocols, are presently in a state of underdeveloped transition. The absence of suitable regulatory standards leads to inconsistency in the application and format of quality control and good manufacturing protocols for biofabricating tumors and metastasis on‐a‐chip. This is, in particular, crucial to the standardization protocols for cell generation, material sourcing and synthesis, and tissue‐informed requisites for safety and efficacy.^113^

**Figure 3:**
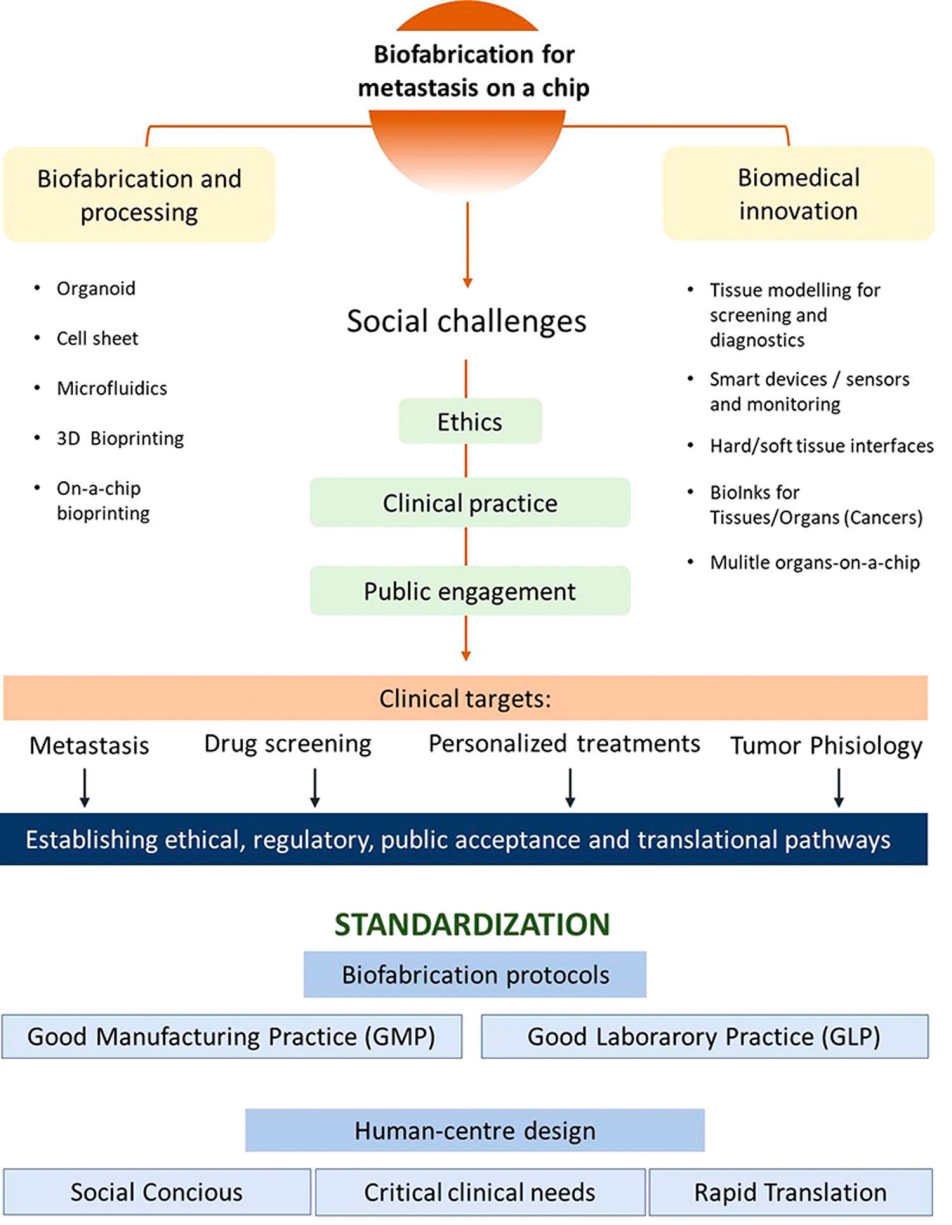
Schematic for the cross‐disciplinary development of biofabrication tumor models on‐a‐chip for use in clinical applications. [Color figure can be viewed at wileyonlinelibrary.com]

In summary, there are well‐defined gaps relative to the fundamentals of life science and the biofabrication technologies, regulatory, standardization, and scalable biomanufacturing protocols for tumor/metastasis on‐a‐chip model that need to be addressed. These gaps need to be resolved before a methodical approach based on human‐centered design can be standardized, established, and translated to clinical trials (Figure 3).

## CONFLICT OF INTEREST

The authors declare no conflict of interest.

## Data Availability

Data sharing is not applicable to this article as no new data were created or analyzed in this study.
